# Grants Recommended by the Distribution Committee

**Published:** 1919-12-20

**Authors:** 


**King Edward's Hospital Fund—(*continued*).**

December 2, 1919.

### Grants Recommended by the Distribution Committee.

*The Distribution Committee desire to draw attention to the fact that it must not be assumed that the reduction or absence of a grant implies dissatisfaction.*

ACTON HOSPITAL, £170. Alexandra Hospital for Children, £200. BECKENHAM COTTAGE HOSPITAL, £60. Belgrave Hospital for Children, £1,125. Blackheath and Charlton Hospital, £145. Bolingbroke Hospital, £1,000. British Hospital for Mothers and Babies, Woolwich, £1,730, of which £1,500 towards the building of the amalgamated hospital, in accordance with the scheme submitted to the Fund. CANNING TOWN WOMEN'S SETTLEMENT HOSPITAL (late Medical Mission), £700, of which £100 to reduce debt. Central London Ophthalmic Hospital, £550. Central London Throat and Ear Hospital, £225. Charing Cross Hospital, £4,600, of which £500 to improvements. Chelsea Hospital for Women, £2,150, of which £1,500 to reduce debt. Cheyne Hospital for Sick and Incurable Children, £400, of which £50 to reduce general fund overdraft. City of London Hospital for Diseases of the Chest (Victoria Park), £4,500, of which £500 to proposed sanitary improvements, in accordance with the scheme submitted to the Fund. City of London Maternity Hospital, £1,550, of which £500 to reduce general fund debt. Clapham Maternity Hospital, £1,025, of which £100 to scheme. DREADNOUGHT HOSPITAL, £3,250. EAST END MOTHERS' LYING-IN HOME, £850. East Ham Hospital, £215. East London Hospital for Children, £2,750. Elizabeth Garrett Anderson Hospital, £1,400, of which £250 to the scheme for the adaptation of the adjoining premises for out-patients and nurses. Eltham and Mottingham Cottage Hospital, £120. FINCHLEY COTTAGE HOSPITAL, £110. Florence Nightingale Hospital for Gentlewomen, £350, of which £100 to reduce debt. French Hospital, £200. GENERAL LYING-IN HOSPITAL, £1,600, of which £100 to reduce debt, and £1,000 to scheme of rebuilding to be submitted to the Fund. Great Northern Central Hospital, £6,100, of which £1,500 to new nurses' home in accordance with scheme to be submitted to the Fund. Grosvenor Hospital for Women, £575. Guy's Hospital, £11,000, of which £2,000 to improvements in nursing accommodation, in accordance with the scheme submitted to the Fund. HAMPSTEAD GENERAL AND NORTH-WEST LONDON HOSPITAL, £3,200, of which £1,500 to reduce debt. Hendon Cottage Hospital, £30. Hornsey Cottage Hospital, £25. Hospital and Home for Incurable Children, Hampstead, £100, of which £50 towards provision of operating theatre. In consideration of the fact that curable cases are admitted. Hospital for Consumption, Brompton (including Sanatorium at Frimley), £7,150. Hospital for Diseases of the Throat, £625, of which £500 towards loss incurred by postponement of building scheme through the war. The Committee trust that the scheme will be pushed forward as rapidly as possible. Hospital for Epilepsy and Paralysis, £2,225, of which £50 to reduce debt. Hospital for Sick Children, £4,600. Hospital for Women (Soho Square), £1,750, of which £250 to reduce debt. Hospital of St. John and St. Elizabeth, £675.

**King Edward's Hospital Fund—(*continued*).**

INFANTS' HOSPITAL, £900, of which £250 to reduce debt. Italian Hospital, £400. KENSINGTON AND FULHAM GENERAL HOSPITAL, £50. Kensington Dispensary and Children's Hospital, £55. King Edward Memorial Hospital (Ealing), £750. King's College Hospital, £8,400, of which £5,000 to reduction of debt on removal to South London, making £77,100 in all contributed by the Fund to this purpose. LEYTON, WALTHAMSTOW, AND WANSTEAD CHILDREN'S AND GENERAL HOSPITAL, £750. London Fever Hospital, £230. London Homœopathic Hospital, £1,950, of which £250 to reduce general fund debt. London Hospital, £13,000, of which £1,000 to deficit on new nurses' home, in accordance with the scheme submitted to the Fund. London Lock Hospital, £4,150, of which £1,050 to maintenance of Harrow Road Hospital, £100 to maintenance of Dean Street Hospital, and £3,000 to reconstruction, in accordance with the scheme submitted to the Fund, making £4,500 granted to the scheme to date. No further grant will be made to the reconstruction until the Fund is satisfied that the appeal to the public is being actively pressed. London Temperance Hospital, £2,300. MATERNITY CHARITY AND DISTRICT NURSES' HOME, PLAISTOW, £350. Memorial Hospital, Mildmay Park, £550, of which £150 towards installation of electric light. Metropolitan Hospital, £4,500, of which £1,000 to reduce debt. Middlesex Hospital, £8,400, of which £500 to reduce debt, and £900 to new nurses' accommodation in accordance with the scheme submitted to the Fund. Middlesex Hospital Cancer Charity, £500, of which £100 to new nurses' accommodation. Mildmay Mission Hospital, £675. Miller General Hospital for South-East London, £4,500, of which £2,500 is a first grant to the extension of the hospital, in accordance with scheme to be submitted to the Fund. Mothers' Hospital of the Salvation Army, £1,000, of which £500 to new nurses' accommodation, in accordance with scheme to be submitted to the Fund. NATIONAL HOSPITAL FOR DISEASES OF THE HEART, £800, of which £400 to extension of out-patient department, in accordance with scheme to be submitted to the Fund. National Hospital for the Paralysed and Epileptic, £4,000. Nelson Hospital (late South Wimbledon, etc.), £550. Norwood Cottage Hospital, £450. PADDINGTON GREEN CHILDREN'S HOSPITAL, £900. Passmore Edwards Hospital for Willesden, £250. Passmore Edwards Hospital for Wood Green, etc., £115. Poplar Hospital for Accidents, £250. Prince of Wales's General Hospital, £6,000, of which £3,000 to reduce general fund debt. QUEEN CHARLOTTE'S LYING-IN HOSPITAL, £2,000, of which £500 to reduce debt, and £700 to deferred scheme of improvements. Queen Mary's Hospital for the East End, incorporating West Ham and Eastern General Hospital, £5,250, of which £1,000 to nurses' home, in accordance with scheme to be submitted to the Fund. Queen's Hospital for Children, £4,500, of which £625 to extinguish debt on old building scheme, and £1,000 to scheme for new out-patient department to be submitted to the Fund. ROYAL DENTAL HOSPITAL OF LONDON, £500. Royal Eye Hospital, £700. Royal Free Hospital, £5,000, of which £1,500 to new nurses' accommodation, in accordance with the scheme submitted to the Fund. Royal Hospital for Diseases of the Chest (City Road), £1,450. Royal Hospital, Richmond, £450. Royal London Ophthalmic Hospital, £2,800, of which £500 to new heating apparatus. Royal National Orthopædic Hospital, £1,250. Royal Waterloo Hospital for Children and Women, £4,250, of which £2,000 to new nurses' accommodation, isolation wards, and extension of out-patient department, in accordance with the scheme submitted to the Fund. Royal Westminster Ophthalmic Hospital, £200. ST. ANDREW'S HOSPITAL (DOLLIS HILL), £310, of which £250 to reduce building debt. St. Columba's Hospital, £600, of which £250 to provision of balconies. St. George's Hospital, £5,000. St. John's Hospital, Lewisham, £675; St. Luke's Hospital for Advanced Cases, £250. St. Mark's Hospital, £550, of which £100 to improvements in nursing accommodation. St. Mary's Hospital, £4,500. St. Mary's Hospital for Women and Children, Plaistow, £3,150, of which £2,000 to the rebuilding of the nurses' and servants' quarters and out-patient department, in accordance with scheme to be submitted to the Fund. St. Monica's Home Hospital, £270, of which £100 to sanitary improvements. St. Peter's Hospital for Stone, £325, of which £200 to new lift. St. Saviour's Hospital for Ladies of Limited Means (Osnaburgh Street), £230. St. Thomas's Hospital, £7,500, of which £2,500 to improvements, in accordance with scheme to be submitted to the Fund. Samaritan Free Hospital for Women, £1,425. Santa Claus Home, £60. South-Eastern Hospital for Children (Sydenham), £660, of which £200 to central heating and laundry improvements. South London Hospital for Women, £750. Stoke Newington Home Hospital for Women, £120. UNIVERSITY COLLEGE HOSPITAL, £6,800, of which £500 to improvements. VICTORIA HOSPITAL FOR CHILDREN, £1,700, of which £250 to central heating. WEST END HOSPITAL FOR NERVOUS DISEASES, £2,750, of which £2,000 to removal to Regent's Park, in accordance with the scheme submitted to the Fund. Western Ophthalmic Hospital, £175. West London Hospital, £6,150, of which £1,500 to improvements, in accordance with scheme to be submitted to the Fund. Westminster Hospital, £4,800, of which £300 annual grant to acquisition of new site as agreed. Wimbledon Hospital, £120. Woolwich and Plumstead Cottage Hospital, £25 (see special grant below).

**SPECIAL GRANT**

| | |
|---|---|
| **Woolwich and District War Memorial Hospital Building Fund ... ...** | **£5,000** |

**SUMMARY.**

| | |
|---|---|
| **Grants ... ... ... ... ... ... ...** | **£213,000** |
| **Special Grant ... ... .. ... ...** | **£5,000** |
| **Total Grants for the Year 1919 ...** | **218,000** |

